# Time boundaries of the three-phase time-sensitive model for ventricular fibrillation cardiac arrest

**DOI:** 10.1016/j.resplu.2021.100095

**Published:** 2021-03-02

**Authors:** Yoshikazu Goto, Akira Funada, Tetsuo Maeda, Yumiko Goto

**Affiliations:** a Department of Emergency and Critical Care Medicine, Kanazawa University Hospital, Takaramachi 13-1, Kanazawa 920-8640, Japan; b Department of Cardiology, Osaka Saiseikai Senri Hospital, Tsukumodai 1-1-6, Suita 565-0862, Japan; c Department of Cardiology, Yawata Medical Center, Yawata I 12-7, Komatsu 923-8551, Japan

**Keywords:** Ventricular fibrillation, Cardiac arrest, Epidemiology, Outcomes, Cardiopulmonary resuscitation

## Abstract

**Aim:**

Ventricular fibrillation (VF) cardiac arrest may consist of three time-sensitive phases: electrical, circulatory, and metabolic. However, the time boundaries of these phases are unclear. We aimed to determine the time boundaries of the three-phase model for VF cardiac arrest.

**Methods:**

We reviewed 20,741 out-of-hospital cardiac arrest cases with initial VF and presumed cardiac origin from the All-Japan Utstein-style registry between 2013 and 2017. The study endpoint was 1-month neurologically intact survival. The collapse-to-shock interval was defined as the time from collapse to the first shock delivery by emergency medical service personnel. The patients were divided into the bystander cardiopulmonary resuscitation (CPR, *n* = 11,606) and non-bystander CPR (*n* = 9135) groups.

**Results:**

In the bystander CPR group, the collapse-to-shock times that were associated with increased adjusted 1-month neurologically intact survival, compared with those in the non-bystander CPR group, ranged from 7 min (42.9% [244/4999] vs. 26.0% [119/458], adjusted odds ratio [aOR], 1.95; 95% confidence interval [CI], 1.44–2.63; *P* < 0.0001) to 17 min (17.1% [70/410] vs. 7.3% [21/288], aOR, 2.82; 95% CI, 1.62–4.91; *P* = 0.0002). However, the neurologically intact survival rate of the bystander CPR group was statistically insignificant compared with that of the non-bystander CPR group when the collapse-to-shock time was outside this range.

**Conclusions:**

The time boundaries of the three-phase time-sensitive model for VF cardiac arrest may be defined as follows: electrical phase, from collapse to <7 min; circulatory phase, from 7 to 17 min; and metabolic phase, from >17 min onward.

## Introduction

Early defibrillation is a crucial factor for neurologically intact survival after ventricular fibrillation (VF) cardiac arrest.[1], [2] However, as the duration of cardiac arrest increases and the pathophysiology of ischaemia/reperfusion progresses over time, uniform immediate defibrillation for all cases of VF cardiac arrest becomes non-optimal.^3^ In 2002, Weisfeldt and Becker^3^ proposed a time-sensitive model for the treatment of VF cardiac arrest, with three phases: electrical, circulatory, and metabolic. According to this model of cardiopulmonary resuscitation (CPR), the optimal treatment for cardiac arrest is phase specific. The potential survival benefit of bystander CPR partly depends on ischaemic time (i.e. time from collapse to return of spontaneous circulation or collapse-to-shock interval), with the greatest benefit achieved during the circulatory phase. However, the time boundaries between phases are not precisely defined in the current literature.

Therefore, we aimed to investigate the relationship among collapse-to-shock time, bystander CPR, and neurologically intact survival, and to determine the time boundaries of the three-phase time-sensitive model for VF cardiac arrest.

## Methods

### Study design and setting

This nationwide, population-based observational study included adult patients aged ≥18 years in Japan for whom resuscitation was attempted after an out-of-hospital cardiac arrest (OHCA) between January 1, 2013, and December 31, 2017. The study was approved by the institutional review board of Kanazawa University (No. 1263). The requirement for written informed consent was waived because the study used anonymised data. In Japan, nearly 127 million individuals reside in an area of approximately 380,000 km^2^. Further, approximately two-thirds of Japan comprises uninhabited mountainous terrain.^4^

The Fire and Disaster Management Agency (FDMA) of Japan supervises a nationwide emergency medical service (EMS) system, whereas local fire stations operate local EMS systems. In 2017, Japan had 732 fire departments and 5140 ambulance teams.^5^ During the study period, all EMS personnel performed CPR following the Japanese CPR guidelines and attempted resuscitation by using automated external defibrillators, inserting airway adjuncts and peripheral intravenous catheters, and administering Ringer's lactate solution.[5], [6], [7] Only specially trained emergency life-saving technicians are permitted to insert tracheal tubes and administer intravenous adrenaline (epinephrine) after receiving online instructions from a physician.^5^ Except in special situations, such as decapitation, incineration, decomposition, rigour mortis, and dependent cyanosis, EMS personnel in Japan are legally prohibited from terminating resuscitation in the field. Most patients with OHCA are administered CPR by EMS personnel before being transported to a hospital.

## Data collection and quality control

In 2005, the FDMA launched an ongoing prospective population-based observational study including all patients with OHCA in Japan who received resuscitation performed by EMS personnel.^5^ EMS personnel and the physician in charge at each centre recorded data from the patients using an Utstein-style recommended guideline template.[8], [9] The data were transferred to individual local fire stations and subsequently integrated into the registry system on the FDMA database server. The database application automatically checked the patient data for consistency, which was further verified by the FDMA. The data were transferred to and stored in a nationwide database that was developed by the FDMA for public use. The FDMA granted the authors permission to access the anonymised data for this study.

The information in the dataset included the following: patient sex and age, aetiology of arrest, initially identified cardiac rhythm, presence of bystander witnesses and their relation to the patient (e.g. family member, layperson other than family, or EMS personnel), manoeuvre of bystander CPR, time of collapse, receipt of emergency calls, time of vehicle arrival at the scene, EMS initiation of CPR, 1-month survival, and neurologically intact survival. The aetiology of arrest was presumed to be cardiac unless suitable evidence suggested a non-medical cause (e.g. trauma, accidental hypothermia, hanging, drowning, drug overdose or poisoning, or asphyxia) or another non-cardiac cause (e.g. respiratory or cerebrovascular disease or malignant tumours). The physicians in charge determined the aetiology of arrest. Neurological outcomes were defined using the Cerebral Performance Category (CPC) scale scores (1: good cerebral performance, 2: moderate cerebral disability, 3: severe cerebral disability, 4: comatose or vegetative state, 5: death).^8^ The CPC scale scores were determined by the physician in charge.

Information on collapse time and presence of bystander interventions was obtained by EMS personnel, who interviewed the bystanders before leaving the scene. All interviews were recorded in a recording medium for the EMS reports, which can be a written record or an audio recording. All time data, including the shock delivery time by EMS personnel, were electronically recorded using a recording device by EMS personnel and/or the EMS centre.

## Study endpoints

The primary study endpoint was neurologically intact survival (CPC scale score = 1 or 2 [CPC 1–2]) at 1 month. The secondary endpoint was 1-month survival after OHCA.

## Statistical analysis

To determine the association of the collapse-to-shock time and bystander CPR with 1-month outcomes from OHCA, we divided the patients into two groups: bystander CPR and non-bystander CPR. Continuous variables are expressed as medians and 25–75 percentiles or as means and standard deviations. Categorical variables are expressed as numbers and percentages. Effect size and variability are reported as odds ratios (ORs) with 95% confidence intervals (CIs).

The Kruskal–Wallis and Dunn's post hoc tests were used to compare continuous variables. The chi-square test was used to compare categorical variables, and univariate logistic regression analysis was performed to compare the characteristics and outcomes between the two groups. Multivariable logistic regression analyses including 10 pre-hospital variables were performed to evaluate the association between the collapse-to-shock interval and 1-month outcomes for all eligible patients. Potential pre-hospital confounders in the analytic model were selected on the basis of biological plausibility and data reported in previous studies. The 10 selected pre-hospital variables included calendar year (as a categorical variable), geographic region in Japan (rural or urban area), age (as a continuous variable), sex (male or female), status of a witness (family member or non-family member), presence of bystander CPR (yes or no), use of advanced airway management (yes or no), adrenaline administration (yes or no), time from collapse to first shock delivery by EMS personnel (collapse-to-shock time, as a continuous variable), and time from the emergency call receipt to EMS arrival at the patient's side (EMS response time, as a continuous variable).

We calculated the crude 1-month outcomes according to the collapse-to-shock time. For each collapse-to-shock time, univariable and multivariable logistic regression analyses were performed to compare the 1-month outcomes between the two groups. All data were analysed using the statistical software package JMP 15-Pro (SAS Institute Inc., Cary, NC, USA). To avoid a high rate of false-positive results and the lack of reproducibility of differences between the two groups, all reported tests were two-tailed and *P* < 0.005 was considered statistically significant.[10], [11]

## Results

**Fig. 1:**
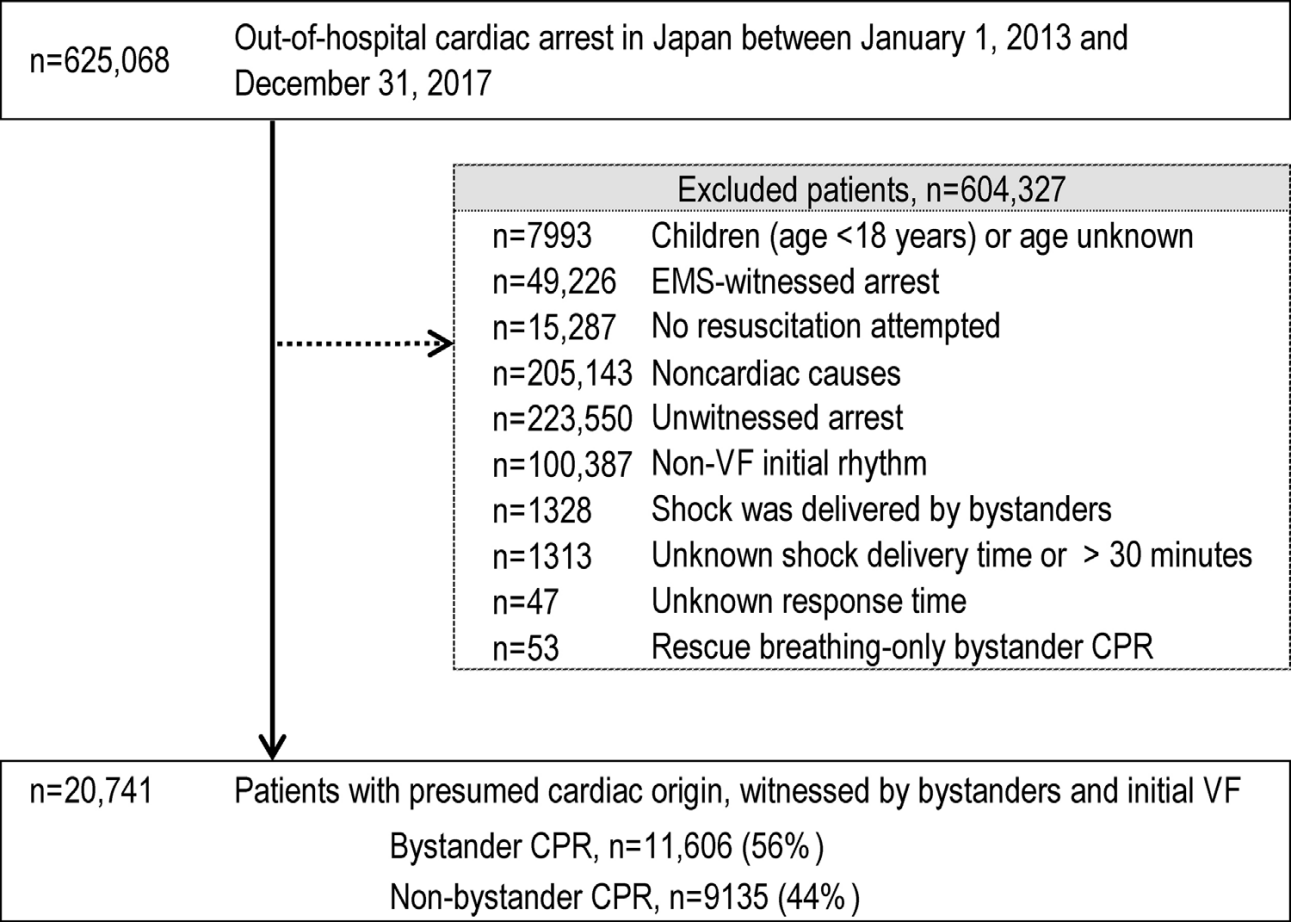
Study inclusion flowchart. CPR, cardiopulmonary resuscitation; EMS, emergency medical services; VF, ventricular fibrillation.

The details of attempted resuscitation for 625,068 patients with OHCA from 2013 to 2017 in Japan were documented in the FDMA database. The inclusion and exclusion criteria of the present study are shown in Fig. 1. Patients with non-cardiac causes of OHCA, with EMS-witnessed arrest, without resuscitation attempted by EMS personnel, aged <18 years, and with unknown outcomes or age were excluded. A total of 20,741 patients (3.3% of all patients in the registry) met the following inclusion criteria and were included in this study: age ≥18 years, presumed cardiac aetiology of arrest, OHCA witnessed by bystanders, arrest with initial VF rhythm, and no public-access defibrillation before the arrival of EMS personnel.

The baseline characteristics of the patients included in this study are shown in Table 1. Patients in the bystander CPR group were more likely to experience OHCA in rural areas, to be younger, and to have an arrest witnessed by a non-family member, and were less likely to receive advanced airway maintenance and adrenaline, than patients in the non-bystander CPR group. The EMS response time, collapse-to-initiation of EMS CPR time, and collapse-to-shock time were significantly longer in the bystander CPR group than in the non-bystander CPR group. The EMS CPR duration before the first shock delivery was significantly shorter in the bystander CPR group than in the non-bystander CPR group. However, the differences between the two groups in those time variables were all within 1 min. The overall crude 1-month outcomes were significantly better in the bystander CPR group than in the non-bystander CPR group.

**Table 1:** Baseline characteristics of the patients.

Characteristic	Bystander CPR		Non-bystander CPR		*P*-value
	*n* = 11,606 (56.0%)		*n* = 9135 (44.0%)		
Year				<0.001	
2013	2168	(18.7)	1987	(21.8)	
2014	2269	(19.6)	1860	(20.4)	
2015	2337	(20.1)	1718	(18.8)	
2016	2425	(20.9)	1811	(19.8)	
2017	2407	(20.7)	1759	(19.2)	
Geographic region in Japan					
Rural area*	2887	(24.9)	1978	(21.7)	<0.001
Age, years					<0.001
Mean (SD)	65.0	(15.6)	66.3	(14.7)	
Median (25–75%)	66	(55–76)	68	(57–77)	
Male sex	9278	(79.9)	7345	(80.4)	0.41
Bystander witness status					<0.001
Family member	7030	(60.6)	5783	(63.3)	
Non-family member	4576	(39.4)	3352	(36.7)	
Advanced airway management	4511	(38.9)	3793	(41.5)	<0.001
Adrenaline administration	3655	(31.5)	3095	(33.9)	<0.001
EMS response time, min					<0.001
Mean (SD)	8.8	(3.1)	8.1	(2.8)	
Median (25–75%)	8	(7–10)	8	(6–10)	
Collapse-to-initiation of EMS CPR time^†^, min					<0.001
Mean (SD)	10.9	(4.6)	9.8	(4.7)	
Median (25–75%)	10	(8–13)	9	(7–12)	
EMS CPR duration before first shock delivery^‡^, min					<0.001
Mean (SD)	1.9	(1.5)	2.2	(1.9)	
Median (25–75%)	2	(1–2)	2	(1–3)	
Collapse-to-shock time, min					<0.001
Mean (SD)	12.4	(4.8)	11.6	(5.1)	
Median (25–75%)	12	(9–15)	11	(8–14)	
1-Month outcome					
Survival	4428	(38.2)	2667	(29.2)	<0.001
CPC 1–2	3237	(27.9)	1632	(17.9)	<0.001

Values are reported as *n* (%) unless indicated otherwise. CPC, Cerebral Performance Category; CPR, cardiopulmonary resuscitation; EMS, emergency medical services; SD, standard deviation.

* Rural area comprises 19 prefectures with a population of <200 inhabitants/km^2^.

^†^Time values were missing for 78 (0.7%) patients in the bystander CPR group and for 282 (3.1%) patients in the non-bystander CPR group.

‡Time values were missing for 2130 (18.4%) patients in the bystander CPR group and for 1311 (14.4%) patients in the non-bystander CPR group.

Supplementary Table S1 shows the adjusted ORs of the pre-hospital variables for 1-month outcomes in the multivariable logistic regression models. Bystander CPR, arrest witnessed by non-family members, non-use of airway management, and non-use of adrenaline were significantly associated with increased odds of 1-month survival and CPC 1–2 rates. Increased collapse-to-shock and EMS response times were associated with significantly decreased 1-month survival and CPC 1–2 rates.

**Fig. 2:**
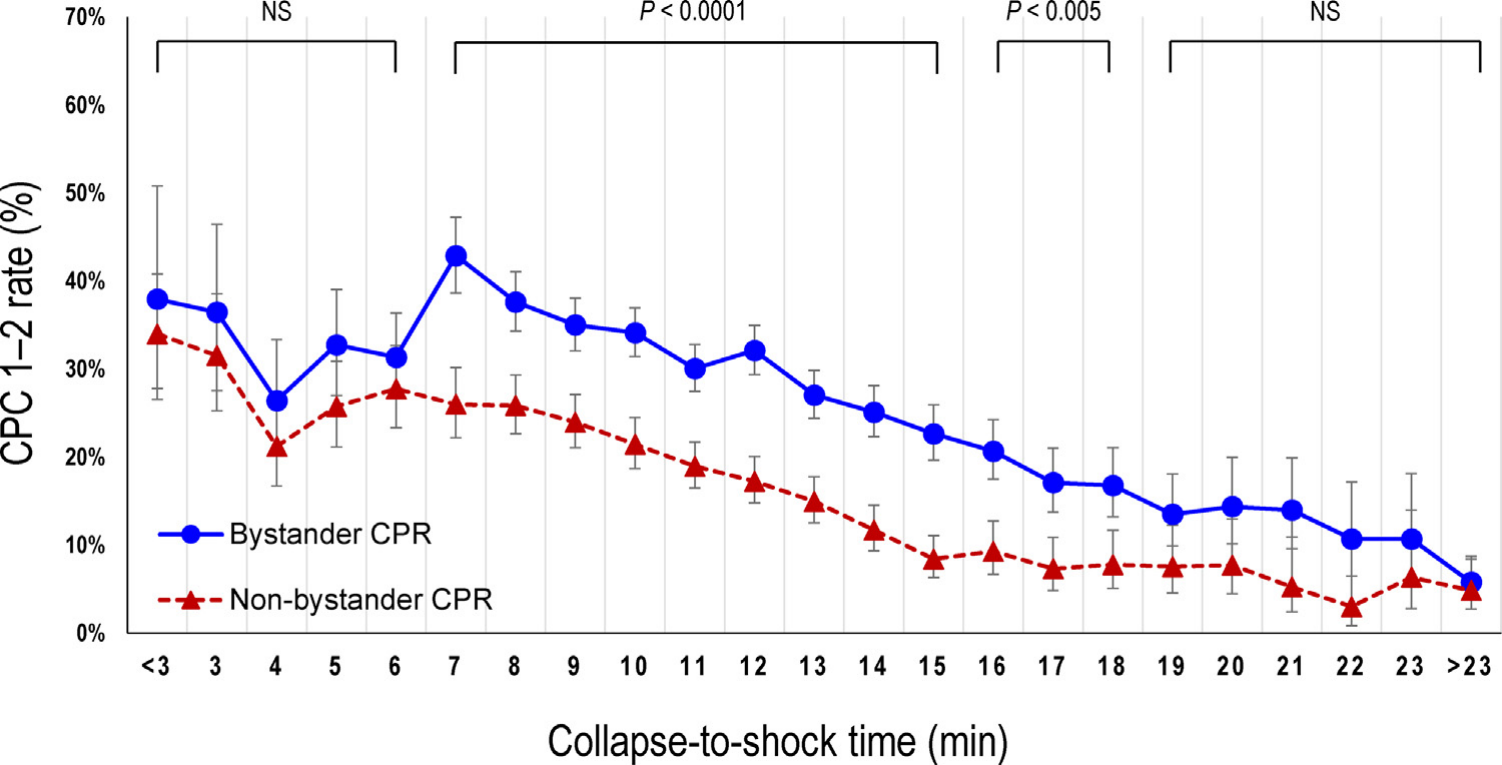
Crude 1-month CPC 1–2 rates according to the collapse-to-shock time. CPC, Cerebral Performance Category; CPR, cardiopulmonary resuscitation; NS, not significant.

**Fig. 3:**
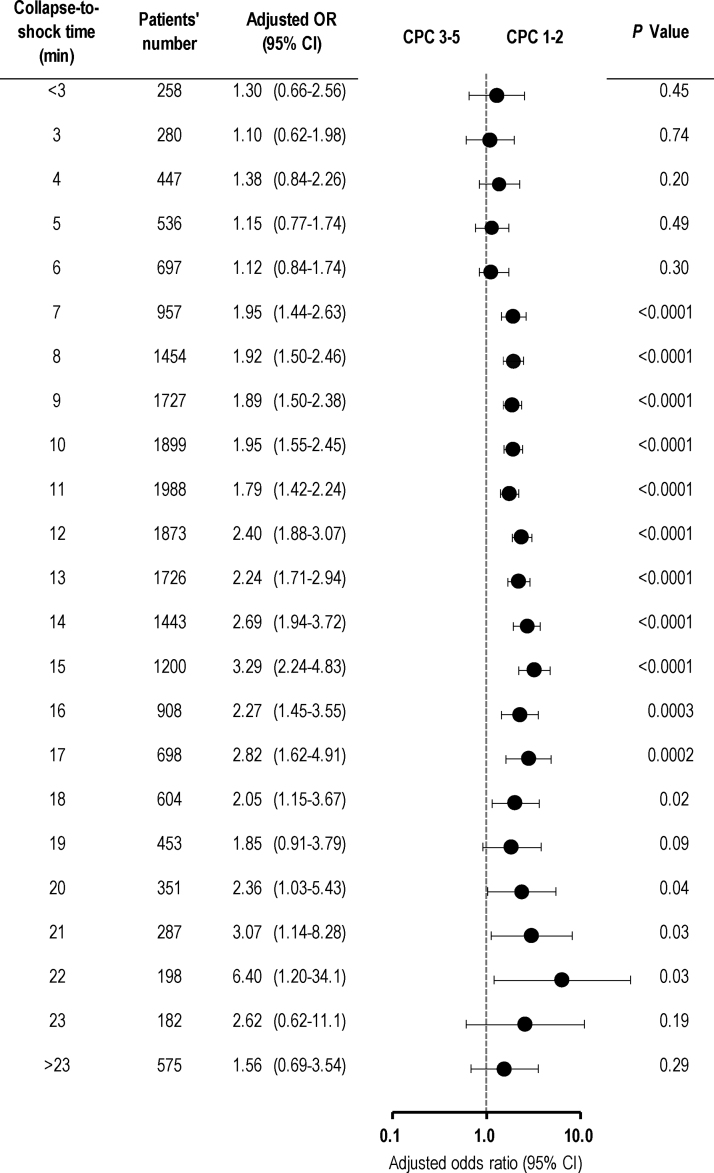
Adjusted odds ratios of bystander CPR for 1-month CPC 1–2 according to the collapse-to-shock time. CI, confidence interval; CPC, Cerebral Performance Category; OR, odds ratio.

The crude 1-month CPC 1–2 rates according to the collapse-to-shock time are shown in Fig. 2. At a collapse-to-shock time of from 7 to 18 min, the crude 1-month CPC 1–2 rates were significantly higher in the bystander CPR group than in the non-bystander CPR group. However, no significant differences in the 1-month CPC 1–2 rates were found between the two groups outside this range. After adjusting for pre-hospital confounders, significant differences in the odds of 1-month CPC 1–2 were found between the two groups at a collapse-to-shock time of from 7 to 17 min (Fig. 3).

**Fig. 4:**
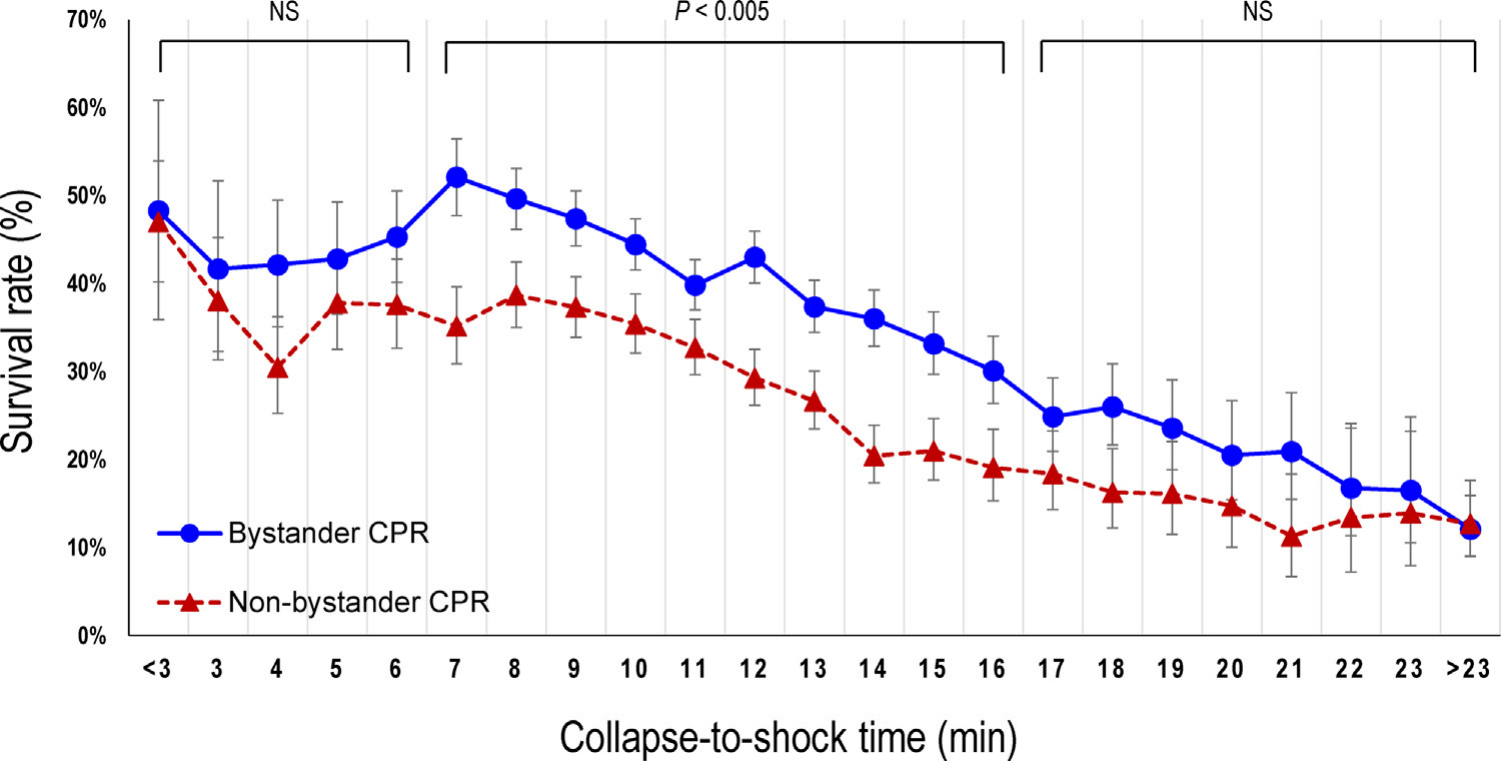
Crude 1-month survival rates according to the collapse-to-shock time. CPR, cardiopulmonary resuscitation; NS, not significant.

**Fig. 5:**
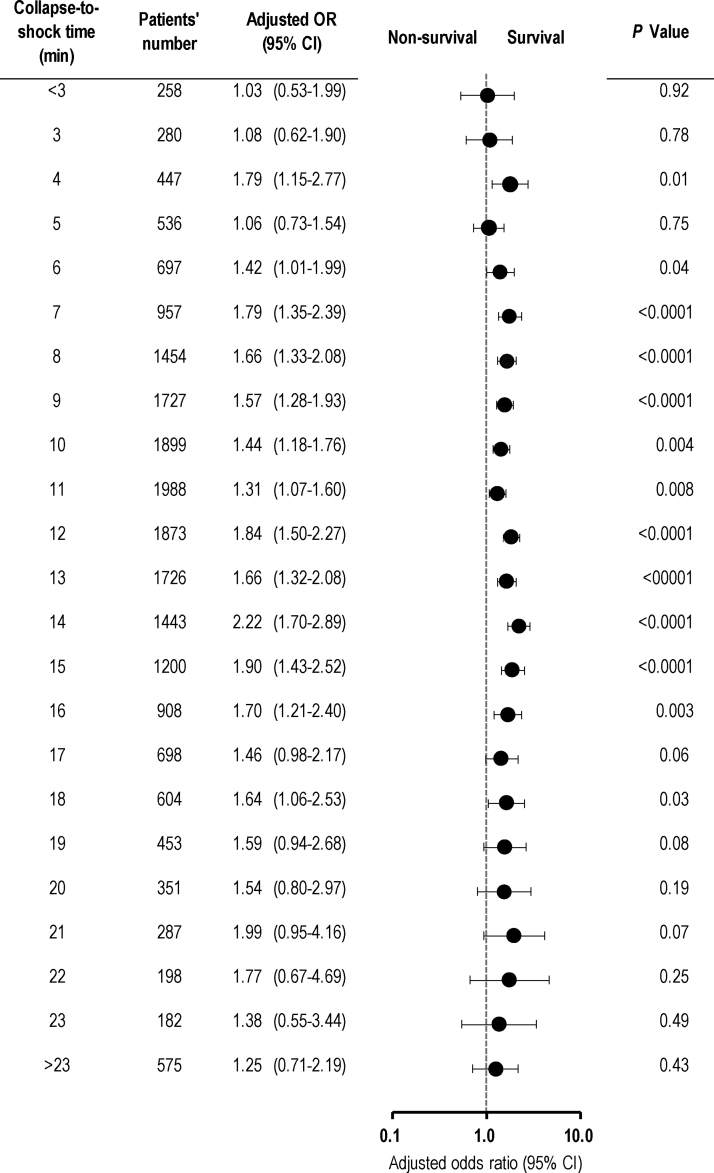
Adjusted odds ratios of bystander CPR for 1-month survival according to the collapse-to-shock time.CI, confidence interval; OR, odds ratio.

The crude 1-month survival rates according to the collapse-to-shock time are presented in Fig. 4. At a collapse-to-shock time of from 7 to 16 min, the crude 1-month survival rates were significantly higher in the bystander CPR group than in the non-bystander CPR group. However, no significant differences in the 1-month survival rate were found between the two groups outside this range. Multivariable logistic regression analysis revealed significant differences in the odds of 1-month survival between the two groups at collapse-to-shock times of from 7 to 10 min and from 12 to 16 min (Fig. 5).

## Discussion

In this nationwide, population-based observational study, we found that the 1-month neurologically intact survival rates in patients with bystander CPR were significantly higher than those in patients with no bystander CPR when the collapse-to-shock time was between 7 and 17 min. However, no significant differences in 1-month neurologically intact survival rates were found between patients with and without bystander CPR when the collapse-to-shock time was <7 or >17 min. These data provide support for the three-phase time-sensitive model for VF cardiac arrest, and suggest that the transition from the electrical to circulatory phase may occur at 7 min and the circulatory phase may extend to 17 min. This is the first large cohort study to clearly demonstrate the time boundaries of the three-phase time-sensitive model for VF cardiac arrest after OHCA.

According to the report by Weisfeldt and Becker,^3^ there are three distinct phases in the time-sensitive model of CPR for VF cardiac arrest: (i) electrical phase, when immediate electrical shock has a high probability of success; (ii) circulatory phase, when chest compressions and ventilation followed by defibrillation might improve the probability of electrical shock success; and (iii) metabolic phase, when a more comprehensive approach to resuscitation would require the establishment of return of spontaneous circulation. However, the time boundaries between phrases are not clearly defined in the previous literature.[3], [12], [13], [14], [15], [16] Gilmore et al.^13^ analysed 2193 patients with OHCA from 1990 to 2004 in the United States, and suggested that the transition from the electrical to circulatory phase may occur at about 5 min (collapse-to-shock time) and the circulatory phase may extend to 15 min. The interval of the electrical phase in the present study was 2 min longer than that in Gilmore et al.’s study. The reasons for this difference may be multifactorial. One reason may be the difference in the CPR guidelines followed by the two studies. In the present study, CPR was performed according to the 2010 and 2015 Japanese CPR guidelines.[6], [7] However, the patients in Gilmore et al.’s study received CPR according to the 1992 and 2000 CPR guidelines.[17], [18] Other reasons may include the quality improvement of bystander CPR^19^ and in-hospital care in Japan.[20], [21] Interestingly, the duration of the circulatory phase was similar between the two studies (10 min). This implies that the benefits of cardiac perfusion by bystander CPR in improving the metabolic state of myocytes may be limited to 10 min, with a potentially more favourable response to defibrillation.

Recent studies have shown that quantitative analysis of features of VF waveform, such as the amplitude spectrum area (AMSA), is generally considered one of the most accurate method of predicting the success of defibrillation.[22], [23], [24], [25] A higher AMSA value has been demonstrated to reflect high myocardial energy stores, which leads to successful progression of defibrillation.^24^ In a porcine model with untreated VF for 10 min followed by 6 min of CPR, AMSA values significantly increased and later defibrillation was successful.^25^ This animal study showed that a VF duration of 16 min was within the circulatory phase, which is consistent with the present study. In the future, prospective studies using quantitative waveform analysis of VF during CPR may more clearly demonstrate the time boundaries of the three-phase time-sensitive model for VF cardiac arrest.

The present study had some limitations. First, the accurate VF duration before shock was not measured in this study. Rather, VF duration was measured as the time from collapse to the first shock delivery, which may have led to an underestimation. Moreover, the number of patients with rhythm conversion from unshockable rhythm to VF by bystander CPR before EMS arrival was unknown. These unmeasured factors may modify the ranges of the three-phase time boundaries. Second, adrenaline administration to patients with sustained VF after the first shock delivery might have affected the three-phase time boundaries. However, we could not analyse these issues in this study. Third, the study analysed data collected from a large national population through standard procedures; however, because of the retrospective observational design, we could not exclude uncontrolled confounders. The present study lacked data on pre-existing comorbidities, location of arrest, quality of bystander- and EMS-initiated CPR, and in-hospital treatments. Further, the present study was also subject to other limitations common to epidemiological studies, including ascertainment bias and lack of data integrity and validity. The relevance of our results to other communities with different emergency care systems and protocols is not known. Similar studies in other countries would help validate our results.

## Conclusions

The time boundaries of the three-phase time-sensitive model for VF cardiac arrest may be as follows: electrical phase, from collapse to <7 min; circulatory phase, from 7 to 17 min; and metabolic phase, from >17 min onward.

## Authors’ contributions

Yoshikazu Goto and Akira Funada designed the study. Yoshikazu Goto, Akira Funada, Tetsuo Maeda, and Yumiko Goto sorted the data. Yoshikazu Goto and Yumiko Goto analysed the data. Yoshikazu Goto drafted the manuscript, and Yumiko Goto and Akira Funada substantially contributed to its revision. Yoshikazu Goto takes responsibility for the paper as a whole. All authors read and approved the final manuscript.

## Credit author statement

All authors have seen and approved the final version of the manuscript being submitted. We warrant that the article is our original work, hasn’t received prior publication and isn’t under consideration for publication elsewhere.

## Funding sources

This work was supported by the 10.13039/501100001691Japan Society for the Promotion of Science (Grant-in-Aid for Scientific Research [B] grant no. 20H202271 and [C] grant no. 18K09999), which had no role in the design and implementation of the study, the analysis and interpretation of the data, or the approval of the manuscript.

## Data statement

The datasets generated and/or analysed during the current study are not publicly available because of the FDMA regulations but are available from the corresponding author upon reasonable request.

## Conflicts of interest

None declared.
