# Supplementary material for: Time boundaries of the three-phase time-sensitive model for ventricular fibrillation cardiac arrest
Source: Resusc Plus. 2021 Mar 2;6:100095. doi: 10.1016/j.resplu.2021.100095 (PMC8244403; doi:10.1016/j.resplu.2021.100095)
Supplement: Supplementary file 1 [file mmc1.docx]

**Supplementary Table**

**Table S1.** Adjusted odds ratios of prehospital variables for 1-month outcomes

| Variables | | Adjusted OR (95% CI) | | | |
| --- | --- | --- | --- | --- | --- |
|  |  | 1-month survival | | 1-month CPC 1 or 2 | |
| Year | |  |  |  |  |
|  | 2014 (vs. 2013) | 1.05 | (0.95–1.16) | 1.00 | (0.89–1.12) |
|  | 2015 (vs. 2013) | 1.13 | (1.02–1.24) | 1.10 | (0.99–1.23) |
|  | 2016 (vs. 2013) | 1.15 | (1.05–1.27) | 1.10 | (0.99–1.23) |
|  | 2017 (vs. 2013) | 1.16 | (1.05–1.27) | 1.07 | (0.96–1.20) |
| Geographic region in Japan | |  |  |  |  |
|  | Rural area (vs. urban area)* | 1.03 | (0.95–1.11) | 1.01 | (0.93–1.10) |
| Age† | | 0.97 | (0.97–0.97) | 0.97 | (0.96–0.97) |
| Male sex (vs. female) | | 0.89 | (0.82–0.96) | 0.94 | (0.85–1.02) |
| Bystander CPR (vs. non-bystander CPR) | | 1.63 | (1.53–1.74) | 2.00 | (1.86–2.15) |
| Witnessed by family member (vs. non-family member) | | 0.94 | (0.88–1.00) | 0.92 | (0.85–0.99) |
| Use of advanced airway management (vs. no use of airway management) | | 0.57 | (0.54–0.61) | 0.45 | (0.42–0.49) |
| Adrenaline administration (vs. no use of adrenaline) | | 0.47 | (0.44–0.51) | 0.35 | (0.32–0.39) |
| Collapse-to-shock time† | | 0.94 | (0.94–0.95) | 0.94 | (0.93–0.95) |
| EMS response time† | | 0.91 | (0.98–0.92) | 0.90 | (0.88–0.91) |
| CI, confidence interval; CPC, Cerebral Performance Category; CPR, cardiopulmonary resuscitation; EMS, emergency medical services; OR, odds ratio. *The rural area is constituted 19 prefectures with population of less than 200 inhabitants per km^2^. †Adjusted odds ratios are reported for 1-year or 1-minute increments. | | | | | |
